# Intraoperative Tumor Migration During Renal Tumor Excision, Veno-Arterial Extracorporeal Membrane Oxygenation (V-A ECMO), and Mechanical Thrombectomy with FlowTriever

**DOI:** 10.7759/cureus.67852

**Published:** 2024-08-26

**Authors:** Arvind Sivashanmugam, Alexander S Doyal, Darvinash Chandra Mohan, Katy Reines, Ricardo A Serrano

**Affiliations:** 1 Medicine, University of North Carolina at Chapel Hill School of Medicine, Chapel Hill, USA; 2 Internal Medicine, Brown University, Providence, USA; 3 Anesthesiology, University of North Carolina at Chapel Hill School of Medicine, Chapel Hill, USA; 4 Urology, University of North Carolina at Chapel Hill, Chapel Hill, USA

**Keywords:** tumor migration, aspiration thrombectomy, flowtriever, intraoperative cardiac arrest, va-ecmo, renal cell carcinoma, pulmonary embolism, transesophageal echo

## Abstract

We describe a case of a 76-year-old male with stage 3 renal cell carcinoma and known thrombus burden in his inferior vena cava (IVC) who presented for a scheduled radical right open nephrectomy with regional lymph node dissection and IVC thrombectomy. During this procedure, the patient went into pulseless-electrical activity. A trans-esophageal echocardiogram showed thrombus transit into the right atria. Emergent initiation of veno-arterial extracorporeal membrane oxygenation and mechanical embolectomy using a FlowTriever retrieval catheter was required. The patient remained intubated in critical but stable condition. Shortly afterward, he expired due to subsequent complications of massive hemorrhage and disseminated intravascular coagulopathy.

## Introduction

Renal cell carcinoma is a common cause of cancer in men and women with an estimated incidence of approximately 81,610 new cases in the United States in 2024 [1]. Renal cell carcinoma has a unique tendency to extend into venous vasculature as a tumor thrombus, with a reported incidence of up to 10% of cases involving the renal vein and the inferior vena cava (IVC) [2]. In 1% of cases, the tumor thrombus can be seen as far as the right atrium [3]. In this situation, an aggressive surgical resection with IVC thrombectomy can lead to higher long-term survival rates. In recent years, different surgical approaches have been implemented to avoid cardiopulmonary bypass [4]. 

When an intraoperative tumor thrombus migrates (clot in transit) into the right heart chambers and cardiac arrest occurs, rapidly deploying a multidisciplinary team to assist the anesthesiologist and surgical team is essential to diagnose and manage this feared and somewhat expected possibility. This case report highlights an approach to one of these critical events and the diagnostic value of the use of trans-esophageal echocardiogram (TEE) monitoring alongside the rapid deployment of veno-arterial extracorporeal membrane oxygenation (V-A ECMO) to support hemodynamics and the intraoperative therapeutic use of newer endovascular mechanical aspiration thrombectomy devices in caring for unstable patients with intraoperative tumor thrombus migration into the right atrium causing interruption of blood flow into the right ventricle.

## Case presentation

**Figure 1:**
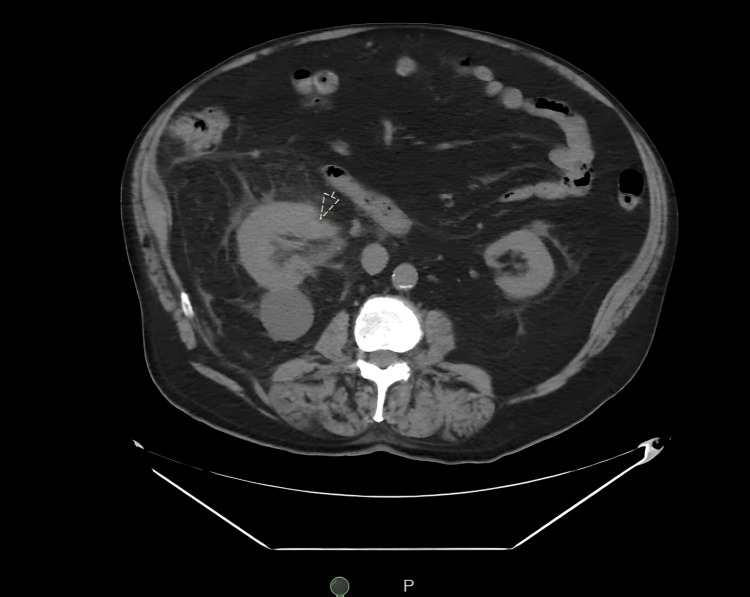
CT Abdomen/Pelvis Image Showing the Right Renal Mass

**Figure 2:**
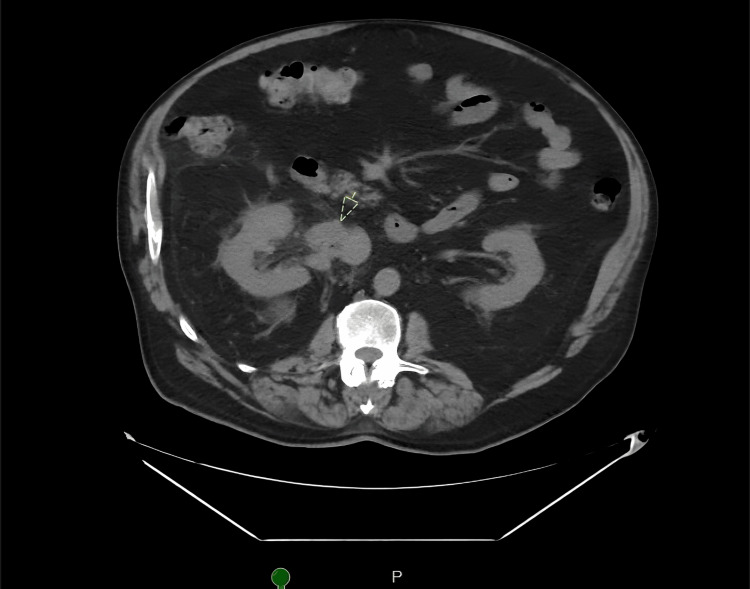
CT Abdomen/Pelvis Image Showing the Right Vein Tumor and IVC Thrombus IVC: Inferior vena cava

A 76-year-old male patient with a history of hypertension and prostate cancer on active surveillance presented to the emergency department with painless hematuria. He also reported decreased appetite and early satiety with a 15-pound weight loss over the prior few weeks. Abdomen and pelvis computed tomography showed a large right renal mass with renal vein and IVC tumor thrombus (Figures 1, 2). Following a multidisciplinary evaluation, including urology and oncology, he was discharged home for outpatient management.

A week later, he presented again to the emergency department with shortness of breath. He was found to have occlusion of the main pulmonary artery with a thrombus that also extended into the right upper lobe, middle lobe, and lower lobe branches, evidenced by computed tomography of his chest. A transthoracic echocardiogram showed a severely dilated and hypokinetic right ventricle. He had an elevated troponin and pro-BNP that suggested cor-pulmonale secondary to the occlusion. The patient was admitted and then underwent a mechanical thrombectomy with rapid improvement of symptoms soon after. He was then therapeutically anti-coagulated and completed a seven-day anticoagulation treatment course of apixaban with plans for indefinite prophylactic dosing. 

**Figure 3:**
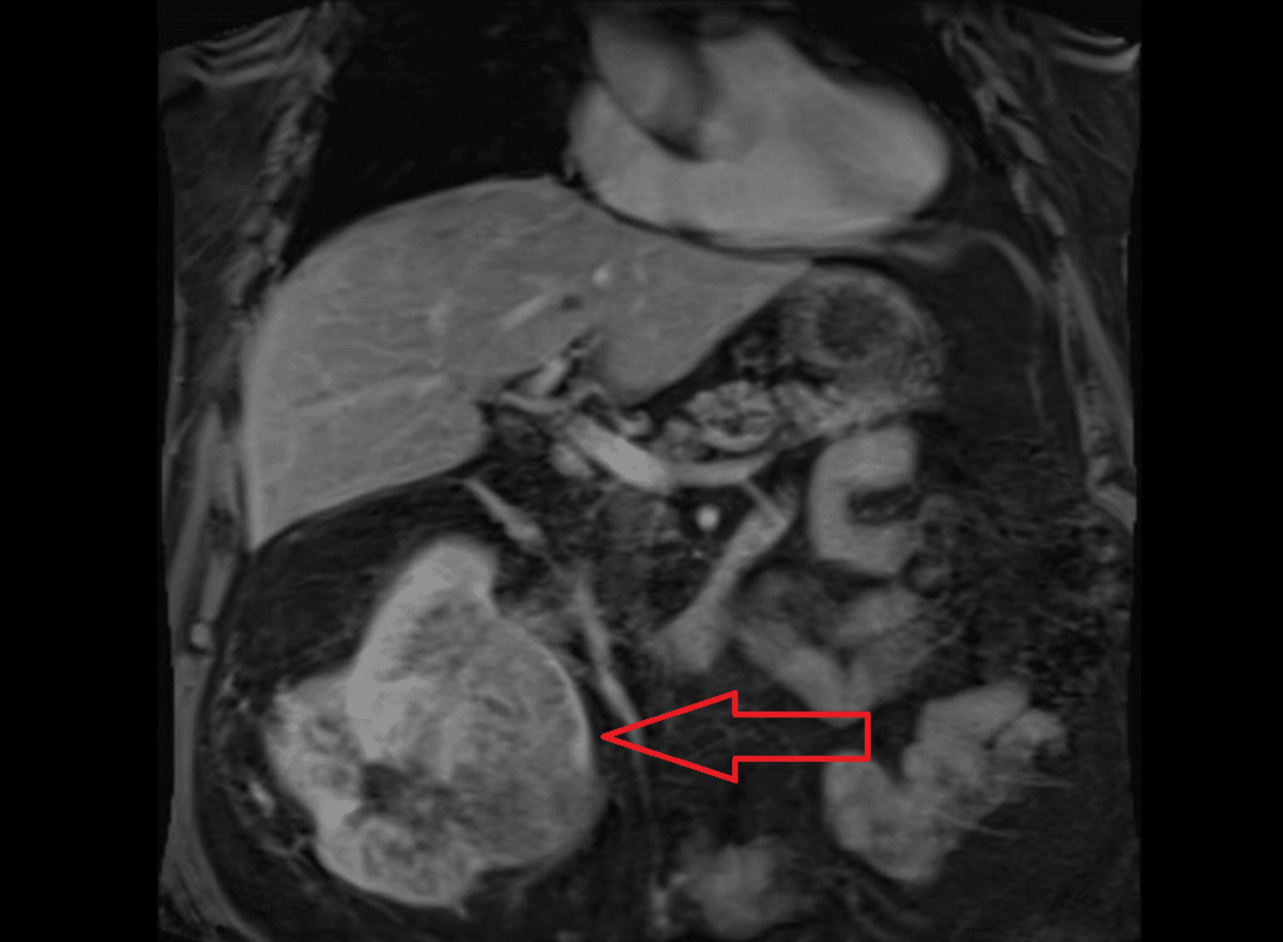
MRI Image Showing Renal Cell Carcinoma

**Figure 4:**
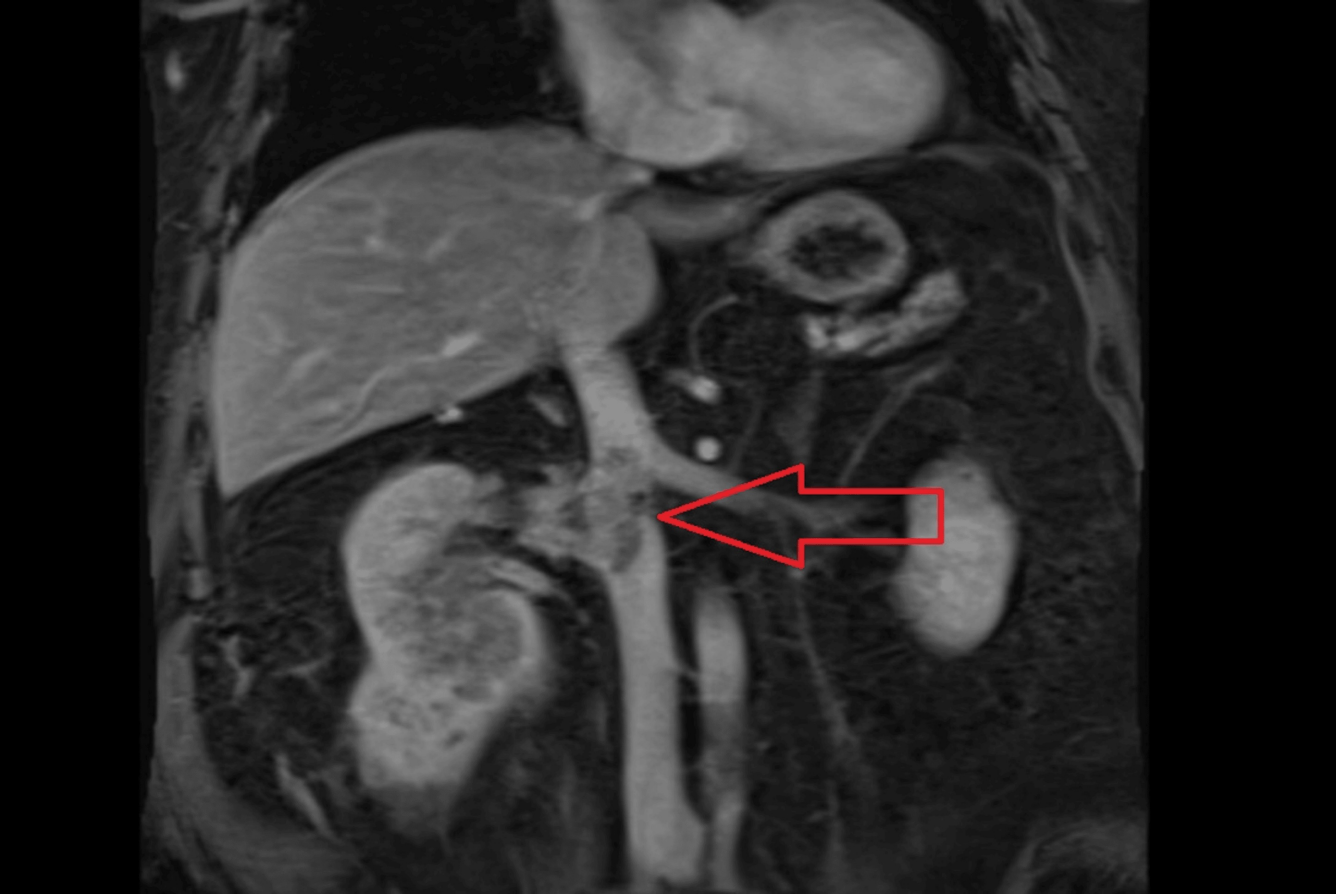
MRI Image Showing IVC Tumor Thrombus IVC: Inferior vena cava

Later, outpatient magnetic resonance imaging of the abdomen for local staging of his suspected cancer showed a large heterogeneous enhancing mass in the lower part of the right kidney invading the renal sinus and right renal vein, with renal vein tumor thrombosis extending into the intrahepatic IVC (Figures 3, 4). 

After multidisciplinary discussion, the patient was scheduled for a radical nephrectomy with IVC thrombectomy and reconstruction. Anesthesia was induced without complications. During the circumferential freeing of the kidney to isolate the hilum, the patient suddenly developed pulseless electrical activity. 

**Figure 5:**
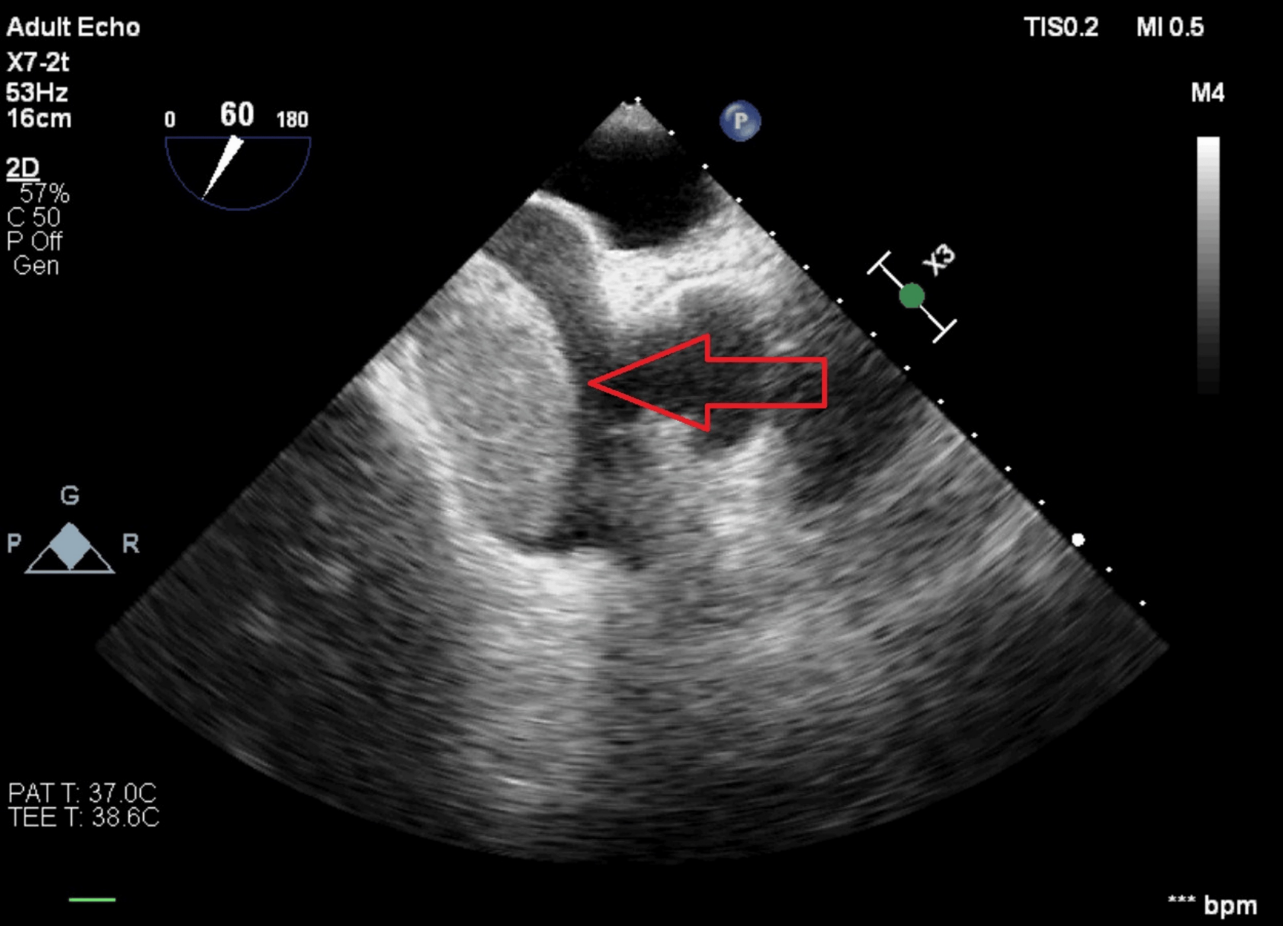
TEE Image Showing the Presence of a Clot in the Right Atria TEE: Trans-esophageal echocardiogram

**Figure 6:**
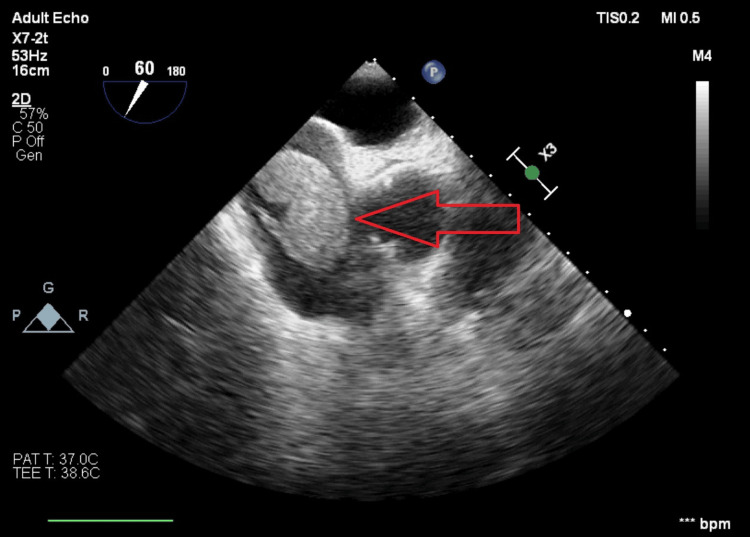
TEE Image Showing the Presence of a Clot in the Right Atria TEE: Trans-esophageal echocardiogram

Chest compressions were immediately initiated, and the TEE showed a thrombus in transit to the right atrium (Figures 5, 6).

**Figure 7:**
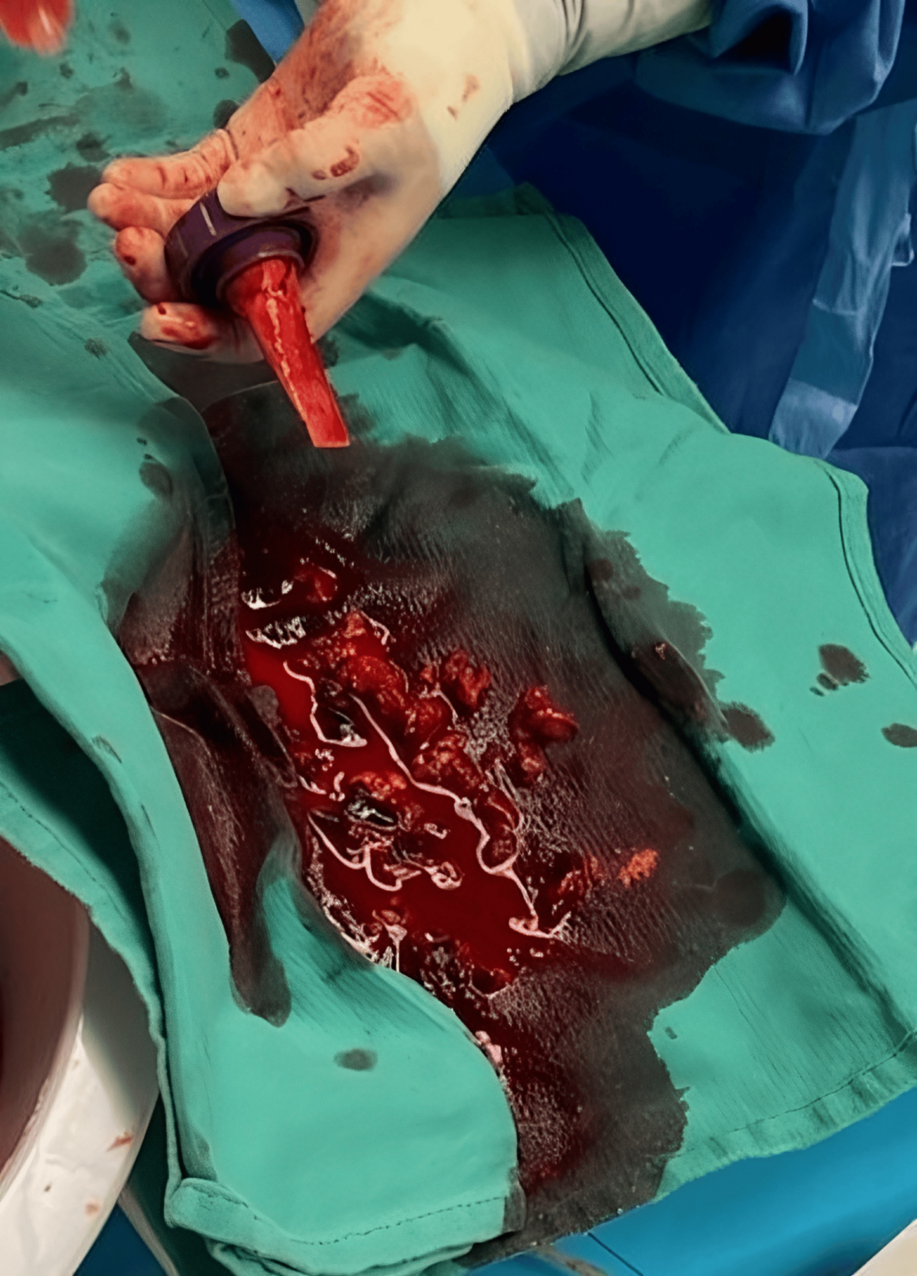
Removed Clot Burden from FlowTriever

Cardiothoracic surgery was immediately involved in performing emergent cannulation for V-A ECMO; at this point, spontaneous circulation was returned. Interventional cardiology was then called into the room to perform mechanical embolectomy with the FlowTriever Aspiration System to remove the tumor thrombus from the right atrium. The thrombus was removed in small fragments with TEE guidance (Figure 7). The renal vein and artery en-bloc were then stapled to free the kidney for removal. The right adrenal gland was also removed. During the mobilization of the liver, subcapsular hematoma and diffuse oozing were noted, resulting in massive hemorrhage and further hemodynamic instability requiring activation of the massive transfusion protocol. Throughout the operation, 25 units of packed red blood cells, 22 units of fresh frozen plasma, prothrombin complex concentrates, two units of cryoprecipitate, two units of platelets, and desmopressin were given. Given the patient's hemodynamic instability and concern for coagulopathy, it was decided not to explore the IVC. The plan was then made to control the surgical bleeding and leave the abdomen open. The patient would then return to the operating room later for abdominal washout and potential V-A ECMO decannulation.

Postoperatively, the patient had progressive hypotension despite vasopressor support, increasing abdominal size, rising lactate, and decreased hemoglobin, suggestive of ongoing intra-abdominal bleeding. During re-exploration, the liver capsule was found to have ruptured with active bleeding from the previously noted subcapsular hematoma. There was also diffuse bleeding from the various raw surfaces with no distinct bleeds or open vessels identified. The diffuse bleeding's etiology was deemed non-surgical as the patient appeared to be in florid disseminated intravascular coagulation (DIC). Despite surgical intervention and transfusion support, the bleeding remained uncontrolled. The poor prognosis was discussed with the family, and the decision was made to withdraw life-sustaining measures, and the patient expired. 

## Discussion

The current case describes an elderly patient with intraoperative cardiovascular collapse secondary to tumor thrombus migration to a position between the right atrium and right ventricle during resection of a large renal cell carcinoma. This required initiation of V-A ECMO, and successful intraoperative mechanical embolectomy with FlowTriever, but with additional complication of significant blood loss with massive transfusion culminating in DIC and death. 

It is suggested that tumor thrombus formation in renal cell carcinoma can be found in approximately 4% to 10% of patients with maybe 1% of them having migration as far as the right atria [5,6]. The surgical approach varies, particularly based on tumor class as defined by the Neves-Zincke classification [7]. This classification is roughly derived from the level of the venous tumor thrombus: Level 1: Renal Vein, Level 2: Infrahepatic IVC, Level 3: Retrohepatic IVC, and Level 4: Right Atrium. Our patient’s tumor extended to the intrahepatic level, falling between Level 2 and Level 3 on this scale. Surgical considerations for the management of renal cell carcinoma with known tumor thrombi at all levels have been under evaluation for many years. Despite moderate complication rates, extracorporeal circulatory support is rare and only appears to be required when tumor thrombus extends into the heart (Level 4) and patients otherwise could be operated on with minimal mortality for those with stable tumor thrombi in the vicinity of the intrahepatic inferior vena cava [8]. Overall, patients undergoing nephrectomy for renal cell carcinoma with known IVC thrombus have an estimated survival rate of 66% at 36 months, but the mortality rate can be as high as 21% if the thrombus extends into the heart [9,10]. There is evidence that mobilizing the liver off the IVC after ligation of the renal vasculature can promote better vascular control and prevent tumor migration [9]. Unique to our case is that the thrombus broke off prior to stapling of the renal vein, which is typically when thrombus migration occurs. 

A previous case has been recently described where it was believed that IVC manipulation led to tumor migration [11].In that report, it was believed to have happened after a clamp was placed on the distal IVC prior to tumor resection. There were no hemodynamic changes in their patient, but the surgical team was no longer able to palpate the tumor thrombus and used TEE monitoring to identify the migration of the thrombus to the right atrioventricular junction. An IVC filter was not explicitly discussed for our case but may have been avoided given the need to manipulate the IVC further for an intervention that is typically temporary. In our literature search, we could not find significant details of any risk factors or protective factors for tumor thrombus migration. Though not well understood, our patient may have been at higher risk for thrombus migration due to his prior history of pulmonary embolism (PE) and new lower extremity edema which may have reflected increased intra-caval thrombus burden than what his prior imaging showed. Ultimately, the case report’s final recommendation was that “intraoperative TEE monitoring should be utilized in resections of renal cell carcinoma with IVC involvement at any level to track thrombus transit and to ultimately improve patient safety”. 

In our case, the patient went into cardiac arrest following moderate IVC manipulation, indicating that the manipulation was the primary cause. Intraoperative TEE was integral in identifying thrombus migration and guiding the FlowTriever System to remove the clot in real time. One unique aspect of this case is the intraoperative use of the FlowTriever System in a patient who is at high risk of tumor thrombus migration into the right-sided heart chambers. The FlowTriever retrieval System was first approved by the FDA for use in PE in 2018, with initial results in small trials showing promising efficacy comparable to surgical embolectomy with seemingly fewer complications [12]. More recently, it was approved in 2021 for the treatment of right atrial clots in transit with only a few reported occurrences for that indication. There continue to be ongoing evaluations in large multicenter trials of the use of FlowTriever or other aspiration thrombectomy devices where it can be an alternative when thrombolysis is contraindicated along with additional benefits of reduced thrombolytic complications and reduced need for postoperative critical care [10]. In our case, where the patient had intraoperative intracardiac tumor migration, there is limited formal guidance on the use of FlowTriever or any other endovascular mechanical thrombectomy device. With the use of FlowTriever, we were able to aspirate a right heart tumor thrombus fully and rapidly with no signs of proceeding right-ventricular strain suggesting displacement nor was there any procedure-related bleeding. The patient had return of spontaneous circulation and while in critical condition, was stable following this intervention. It should be noted that while likely not to have caused bleeding given close visualization during the procedure, the FlowTriever does not recirculate aspirated blood and could have exacerbated hypovolemia in this patient [13]. 

Despite favorable outcomes regarding the thrombus with aspiration thrombectomy, it cannot be ignored that these patients tend to be at increased risk for acute cardiac decompensation. Our case also highlights the utility of V-A ECMO as a tool to temporarily support these patients while addressing underlying causes. Performing aspiration thrombectomy without V-A ECMO may lead to worsening hemodynamics and eventual demise, but there is still high risk of hemodynamic compromise even without ECMO support [14,15]. This is reiterated in studies on patients having received mechanical thrombectomy, with the additional suggestion that monitoring pulmonary artery systolic pressure may be helpful in better risk stratifying those likely to decompensate [16]. Preoperative imaging with point-of-care ultrasound in this patient showed no obvious changes in their tumor burden, so PA pressures in conjunction with intraoperative imaging may be more useful empirically to better asses any possible migration of Level 4 thrombi given the speed at which it can occur. 

## Conclusions

This case emphasizes the necessity of multidisciplinary care for monitoring patients with known tumor thrombus, especially in anticipation of any procedure. The risk factors for tumor migration are unclear, but the use of the TEE can guide rapid intervention and avoid immediate mortality. Aspiration thrombectomy appears to be reasonable management for intracardiac tumor migration, but further studies are needed to establish formal guidance from surgical societies. 
